# A New Predictive Model for the Prognosis of MDA5^+^ DM-ILD

**DOI:** 10.3389/fmed.2022.908365

**Published:** 2022-06-15

**Authors:** Qian Niu, Li-qin Zhao, Wan-li Ma, Liang Xiong, Xiao-rong Wang, Xin-liang He, Fan Yu

**Affiliations:** Department of Respiratory and Critical Care Medicine, Union Hospital, Tongji Medical College, Huazhong University of Science and Technology, Wuhan, China

**Keywords:** interstitial lung disease, anti-MDA5, dermatomyositis, prognosis, nomogram

## Abstract

**Purpose:**

The purpose of this study is to analyze clinical information and combine significant parameters to generate a predictive model and achieve a better prognosis prediction of dermatomyositis-associated interstitial lung disease with positive melanoma differentiation-associated gene 5 antibody (MDA5^+^ DM-ILD) and stratify patients according to prognostic risk factors appropriately.

**Methods:**

We retrospectively reviewed 63 patients MDA5^+^ DM-ILD who were treated in our hospital from January 2018 to January 2021. Our study incorporated most clinical characteristics in clinical practice to explore the associations and predictive functions of clinical characteristics and prognosis. Student's *t*-test, Mann-Whitney *U*-test, chi-squared test, Pearson correlation analysis, Cox regression analysis, R, receiver operating characteristic curves (ROC curves), and Kaplan-Meier survival curves were performed to identify independent predictors for the prognosis of MDA5^+^DM-ILD.

**Results:**

In all the 63 patients with MDA5^+^DM-ILD, 44 improved but 19 did not. Poor prognosis was found more frequently in patients who were older, clinically amyopathic variant of dermatomyositis (CADM), and/or with short duration, short interval of DM and ILD, long length of stay, fever, dyspnea, non-arthralgia, pulmonary infection, pleural effusion (PE), high total computed tomography scores (TCTs), ground-glass opacity (GGO), consolidation score, reticular score and fibrosis score, decreased forced vital capacity (FVC), forced expiratory volume in 1s (FEV1), albumin, A/G, glomerular filtration rate (GFR) and tumor necrosis factor α (TNFα), high titer of anti-MDA5, proteinuria, high levels of monocyte, lactate dehydrogenase (LDH), ferritin (FER), neuron specific enolase (NSE) and glucocorticoid, antibiotic, antiviral, and non-invasive positive pressure ventilation (NPPV). The multivariate Cox regression analysis demonstrated that duration, fever, PE, TCTs and aspartate transaminase (AST) were independent predictors of poor prognosis in patients with MDA5^+^DM-ILD. The nomogram model quantified the risk of 400-day death as: duration ≤ 4 months (5 points), fever (88 points), PE (21 points), TCTs ≥10 points (22 points), and AST ≥200 U/L (100 points) with high predictive accuracy and convenience. The ROC curves possessed good discriminative ability for combination of fever, PE, TCTs, and AST, as reflected by the area under curve (AUC) being.954, 95% CI 0.902–1.000, and sensitivity and specificity being 84.2 and 94.6%, respectively.

**Conclusion:**

We demonstrated that duration, fever, PE, TCTs, and AST could be integrated together to be independent predictors of poor prognosis in MDA5^+^ DM-ILD with highly predictive accuracy.

## Introduction

Dermatomyositis (DM), a multisystem autoimmune disease and a common subtype of idiopathic inflammatory myopathy (IIM), attracts attention from the medical field. In addition to typical skin and muscle involvement, respiratory, digestive, and circulatory system damage, and even malignant tumor can complicate DM. CADM accounts for ~20% of all DM cases. Approximately 87% of MDA5^+^ DM-ILD cases fulfilled Sontheimer's CADM criteria in a Chinese multi-centered cohort (1). ILD, with an incidence of 5–80% and a high risk in positive ARS antibodies and Black ethnicity, is one of the important respiratory lesions in patients with DM (2). Overall, the prognosis of ILD in IIM is good: 50–66% may be expected to have a stable disease course over a substantial period of time. Frustratingly, the remaining proportion will show signs of worsening lung disease within 12 months.

MDA5, a cytoplasmic RNA helicase belonging to the retinoic acid-inducible gene-I (RIG-I) family, which can recognize ds-RNAof viruses and plays an important role in the innate immune system during RNA viral infections, has been identified as a DM-specific autoantigen (3, 4). Anti-MDA5, a 140-kDa polypeptide and one of the myositis-specific autoantibodies named after its autoantigen, was first found in 2005 by immunoprecipitation in Japanese patients (5). The incidence of MDA5^+^ DM ranges from 10 to 20% in Japan, 17.6–22.6% in China, and 7–13% in the United States (6–9). The cumulative 100-month survival rate for the entire patients with MDA5^+^ DM is 66%, and fatal outcomes occur remarkably often within the first 6 months of the diagnosis (10). Patients who responded to therapy and survived had a significantly lower mean titer of anti-MDA5, which was significantly decreased down to below the cutoff level after treatment, while those who did not respond and died had a high level of anti-MDA5 (7, 11), indicating from the side that anti-MDA5 titer is also useful for evaluation of treatment response.

Patients with DM with anti-MDA5 are prone to develop ILD, with a probability of 50–100% (8, 12, 13). Current views regard anti-MDA5 level as a novel parameter for monitoring disease activity and a good predictor of rapidly progressing ILD (RP-ILD) and decreased survival in patients with DM or CADM (11, 14). Early cohort studies reported a high 6-month mortality varying from 33 to 66% in MDA5^+^DM-ILD (10, 15, 16). A multivariate logistic analysis reported by Chen et al. (9) showed that anti-MDA5 is an independent risk factor for death in DM-ILD. Previous studies on the predictive role of clinical characteristics for MDA5^+^DM-ILD are relatively limited. For instance, the relationship between serum ferritin level and abnormality of T cell counts and the disease activity of RP-ILD was reported (17). As the increase of both the morbidity and mortality in MDA5^+^ DM-ILD and the etiology and pathogenesis remaining unknown, early recognition of risk factors for death is particularly important. The aim of this research project is, therefore, to try and establish a meritorious predictive model of prognosis in MDA5^+^ DM-ILD.

## Materials and Methods

### Patients and Inclusion Criteria

We retrospectively reviewed all patients with MDA5^+^DM-ILD from the Department of Rheumatology and the Department of Respiratory and Critical Care Medicine between January 2018 and January 2021 who fulfilled the Bohan and Peter (18, 19) myositis criteria for DM or the Sontheimer (20) criteria for CADM and ILD imaging features. A total of 63 patients were identified. Clinical characteristics consisted of basic information, prognosis, clinical symptoms and signs, complications, treatment means, imaging information, pulmonary functions, and laboratory examinations. We followed all the enrolled patients, and the primary outcome of interest was mortality during the 400-day follow-up.

### Acquisition and Analysis of Computed Tomography Imaging

All CT scans were obtained in the supine position using one of the following scanners: SOMATOM Perspective, SOMATOM Spirit, or SOMATOM Definition AS+ (Siemens Healthineers, Forchheim, Germany). Scans were conducted from the level of the upper thoracic inlet to the inferior level of the costophrenic angle, and images were reconstructed with a slice thickness of 1 or 1.5 mm.

For each patient, predominant CT patterns such as GGO, consolidation, reticulation, emphysema, thickening of the adjacent pleura, pleural effusion, presence of nodules or masses, honeycombing, bronchiectasis, and interlobar pleural traction were independently reviewed by two experienced observers according to the Fleischner Society glossary (21). CT evidence of fibrotic-like changes was defined as the presence of traction bronchiectasis, parenchymal bands (22), and/or honeycombing (21, 23, 24). To quantify the extent of pulmonary abnormalities (GGO, consolidation, reticulation, and fibrotic-like changes), a semiquantitative CT score (25) was assigned on the basis of the area involved in each of the five lung lobes (right upper, middle, and lower, and left upper and lower lobes): 0, no involvement; 1, <5%; 2, 5–25%; 3, 26–49%; 4, 50–75%, and 5, >75%. Total CT score was calculated by summing the individual lobar scores (possible scores range from 0 to 25).

### Pulmonary Function Test

The patients underwent standard pulmonary function testing (PFT) including ventilatory function and diffusion function using Pulmonary Function Testing System (MasterScreen, CareFusion Germany 234 GmbH or Vyaire Medical GmbH) with indoor temperature 24°C, relative humidity 50–70%, and standard atmospheric pressure 760 mmHg. Among all tested indexes, we had principally concentrated on FVC, FEV1, FEV1/FVC, and diffusion capacity of the lung for carbon monoxide (DL_CO_). The results were normalized with age-, sex-, height-and weight-matched control subjects.

### Anti-MDA5 Examination

Serum samples were routinely collected from the patients at initial hospitalization. Anti-MDA5 was detected using commercially available kits (EUROIMMUN, Lübeck, Germany) by Guangzhou Oumeng Medical Laboratory, with a positive control provided in the kit and a negative control provided in the buffer. The criteria for interpretation of results were based on the staining degree of antigen band recognized automatically with EUROBlotOne (EuroImmun, Lübeck, Germany): negative (–) for colorless, doubtful [(+)] for very weakly colored, weakly positive (+) for weakly colored, positive (++) for strongly colored, and strongly positive (+++) for the same intensity with the quality control blot.

### Statistical Analysis

Continuous variables were presented as the mean with standard deviation and categorical variables were expressed as frequency with percentage, and differences between clinical characteristics and prognosis were compared by Student's *t*-test or Mann-Whitney *U*-test and chi-squared test. Significant variables were selected for Pearson correlation analysis and univariate Cox regression analysis. Significant (*P* <0.05) and clinically focused variables in the univariate Cox regression analysis were selected for further multivariate Cox regression analysis. Regression coefficients were regarded as weights for the variables in ROC curves. The nomogram applied to create the scoring system was developed with independent risk factors based on multivariate Cox regression analysis using the “*rms*” package in R. Survival rates were calculated using the Kaplan-Meier method. A two-sided *P*-Value <0.05 was defined as statistically significant. All the analyses were performed using SPSS 25.0 and GraphPad Prism 8.0.2.

## Results

### Patients and Clinical Characteristics

Among the 63 patients with MDA5^+^DM-ILD admitted in our hospital between January 2018 and January 2021, 44 survived and improved, but 19 lost their lives during the 400-day follow-up. The 400-day mortality in our data of all the 63 patients with MDA5^+^DM-ILD was 30.16%. The clinical characteristics are summarized in Tables 1–4, Supplementary Table 1 based on prognosis. Of the 19 patients who died during follow-up, 11 (57.9%) and 8 (42.1%) were confirmed to be anti-MDA5-positive and strongly positive, respectively. The group included nine women (47.4%) and 10 men (52.6%) with a median age of 56.95 years (range 40–68) and mean TCTs of 23.21 (range 6–54), and 2 (10.5%) being smoker, and 11 (57.9%) being CADM. Of the 44 patients who improved, 7 (15.9%), 14 (31.8%), and 23 (52.3%) were confirmed to be anti-MDA5 weakly positive, positive, and strongly positive, respectively. This group included 28 women (63.6%) and 16 men (36.4%) with a median age of 45.8 years (range 19–72) and mean TCTs of 10.02 (range, 0–39), and 8 (18.2%) being smoker and 11 (25%) being CADM.

**Table 1:** Basic information of MDA5^+^DM-ILD.

**Characteristics**	**Total (*N* = 63)**	**Not improved (*n* = 19)**	**Improved (*n* = 44)**	***P*-value**
Age, years	49.16 ± 12.16	56.95 ± 7.81	45.80 ± 12.22	0.000
Sex, male/female	26/37	10/9	16/28	0.229
Ever smoker, *n* (%)	10 (15.9)	2 (10.5)	8 (18.2)	0.698
CADM, *n* (%)	22 (34.9)	11 (57.9)	11 (25.0)	0.012
Duration, m	7.83 ± 14.53	2.87 ± 3.50	9.97 ± 16.85	0.010
Interval of DM and ILD, m	5.47 ± 13.63	1.16 ± 3.40	7.33 ± 15.85	0.017
Length of stay, days	15.11 ± 9.89	21.21 ± 15.29	12.48 ± 4.44	0.024
Fever, *n* (%)	34 (54.0)	18 (94.7)	16 (36.4)	0.000
Cough, *n* (%)	39 (61.9)	15 (78.9)	24 (54.5)	0.067
Dyspnea, *n* (%)	40 (63.5)	18 (94.7)	22 (50.0)	0.001
Arthralgia, *n* (%)	46 (73.0)	10 (52.6)	36 (81.8)	0.017
Myalgia or myasthenia, *n* (%)	49 (77.8)	14 (73.7)	35 (79.5)	0.854
Skin ulcer, *n* (%)	23 (36.5)	4 (21.1)	19 (43.2)	0.094
Gottron sign, *n* (%)	23 (36.5)	7 (36.8)	16 (36.4)	0.971
Helicotrop rash, *n* (%)	43 (68.3)	10 (52.6)	33 (75.0)	0.080
Raynaud phenomenon, *n* (%)	9 (14.3)	2 (10.5)	7 (15.9)	0.867
Pulmonary infection, *n* (%)	35 (55.6)	19 (100.0)	16 (36.4)	0.000
Pleural effusion, *n* (%)	23 (36.5)	14 (73.7)	9 (20.5)	0.000
Subcutaneous emphysema, *n* (%)	1 (1.6)	1 (5.3)	0 (0.0)	0.302
Mediastinal emphysema, *n* (%)	20 (3.2)	1 (5.3)	1 (2.3)	0.516
Pleural thickness, *n* (%)	17 (27.4)	4 (21.1)	13 (30.2)	0.486
Internal malignancy, *n* (%)	0 (0.0)	0 (0.0)	0 (0.0)	
Glucocorticoid, *n* (%)	63 (100.0)	19 (100.0)	44 (100.0)	
Glucocorticoid, mg	159.02 ± 208.45	326.32 ± 284.65	86.77 ± 105.02	0.002
Immunosuppressor, *n* (%)	40 (63.5)	11 (57.9)	29 (65.9)	0.544
Antibiotic, *n* (%)	48 (76.2)	19 (100.0)	29 (65.9)	0.010
Antiviral, *n* (%)	17 (27.0)	11 (57.9)	6 (13.6)	0.000
Anti-fibrosis, *n* (%)	21 (33.3)	8 (42.1)	13 (29.5)	0.332
NPPV, *n* (%)	12 (19.0)	12 (63.2)	0 (0.0)	0.000
Survival time, days	298.89 ± 152.45	102.00 ± 95.27	400.00 ± 0.00	0.000

*The dosage of glucocorticoid was changed to that of methylprednisolone. Immunosuppressors included ciclosporin, cyclophosphamide, tripterygium wilfordii glycosides, mycophenolate mofetil, thalidomide, leflunomide, tacrolimus, and methotrexate. Antifibrotic drugs referred to nintedanib and pirfenidone. CADM, clinically amyopathic variant of dermatomyositis; DM, dermatomyositis; ILD, interstitial lung disease; NPPV, non-invasive positive pressure ventilation*.

**Table 2:** Pulmonary examinations for MDA5^+^DM-ILD.

**Characteristics**	**Total (*N* = 63)**	**Not improved (*n* = 19)**	**Improved (*n* = 44)**	***P*-value**
TCTs	14.06 ± 12.49	23.21 ± 14.14	10.02 ± 9.28	0.001
GGO score	5.05 ± 4.51	7.32 ± 5.19	4.05 ± 3.83	0.007
Consolidation score	3.05 ± 4.25	6.63 ± 5.27	1.47 ± 2.43	0.001
Reticular score	2.76 ± 3.42	4.26 ± 3.77	2.09 ± 3.06	0.020
Fibrosis score	3.23 ± 4.20	5.05 ± 5.02	2.42 ± 3.56	0.022
FVC (L)	2.60 ± 0.88	2.12 ± 0.52	2.75 ± 0.92	0.022
FEV1 (L)	2.09 ± 0.68	1.75 ± 0.33	2.19 ± 0.72	0.022
FEV1/FVC	81.18 ± 9.40	83.91 ± 12.14	80.37 ± 8.53	0.357
DL_CO_ (mmol/min/kPa)	4.92 ± 2.25	4.06 ± 2.26	5.18 ± 2.23	0.253

*TCTs, total CT score; GGO, ground-glass opacity; FVC, forced vital capacity; FEV1, forced expiratory volume in 1s; DL_CO_, diffusion capacity of the lungs for carbon monoxide*.

**Table 3:** General laboratory tests for MDA5^+^DM-ILD.

**Characteristics**	**Total (*N* = 63)**	**Not improved (*n* = 19)**	**Improved (*n* = 44)**	***P*-value**
Leukocyte, G/L	5.56 ± 2.83	6.61 ± 4.28	5.10 ± 1.79	0.052
Monocyte, G/L	0.41 ± 0.20	0.53 ± 0.20	0.35 ± 0.17	0.001
Monocyte, %	7.91 ± 3.64	8.95 ± 3.02	7.47 ± 3.82	0.140
Neutrophil, G/L	4.24 ± 2.67	5.20 ± 4.01	3.82 ± 1.72	0.059
Neutrophil, %	73.75 ± 11.24	76.11 ± 8.02	72.73 ± 12.32	0.202
Lymphocyte, G/L	0.83 ± 0.42	0.78 ± 0.32	0.85 ± 0.45	0.581
Lymphocyte, %	16.78 ± 9.11	13.70 ± 7.18	18.11 ± 9.60	0.078
**Blood urine**				
Negative, *n* (%)	44 (75.9)	9 (56.3)	35 (83.3)	0.070
Positive, *n* (%)	14 (24.1)	7 (43.8)	7 (16.7)	
**Proteinuria**				
Negative, *n* (%)	36 (62.1)	6 (37.5)	30 (71.4)	0.017
Positive, *n* (%)	22 (37.9)	10 (62.5)	12 (28.6)	
AST, U/L	94.48 ± 121.17	150.63 ± 161.88	70.23 ± 90.58	0.054
ALT, U/L	70.63 ± 103.17	94.63 ± 84.83	60.02 ± 109.54	0.226
LDH, U/L	371.81 ± 145.23	458.05 ± 142.07	333.71 ± 130.89	0.001
Alb, g/L	32.68 ± 4.74	29.31 ± 3.77	34.14 ± 4.39	0.000
Glb, g/L	27.68 ± 5.05	27.27 ± 3.25	27.85 ± 5.68	0.615
A/G	1.22 ± 0.29	1.08 ± 0.21	1.28 ± 0.30	0.006
CK, U/L	201.30 ± 300.15	236.26 ± 374.21	185.48 ± 263.69	0.545
GFR, ml/(min/1.73 m^2^)	111.65 ± 21.96	100.95 ± 28.74	115.76 ± 17.49	0.025
Bun, mmol/L	4.47 ± 3.21	5.81 ± 5.32	3.89 ± 1.37	0.137
Cr, μmol /L	62.40 ± 80.92	84.87 ± 146.37	52.69 ± 12.36	0.351
ESR, mm/h	34.67 ± 23.69	38.56 ± 24.57	33.05 ± 23.42	0.412
CRP, mg/L	16.80 ± 27.58	31.72 ± 44.49	10.26 ± 10.85	0.059
FER, μg /L	1,082.04 ± 870.39	1,512.62 ± 1,125.17	866.75 ± 623.90	0.033
CEA, μg /L	7.05 ± 5.39	8.74 ± 7.13	6.26 ± 4.25	0.142
CYFRA, ng/ml	5.70 ± 8.19	9.54 ± 12.23	3.40 ± 2.63	0.075
SCCA, ng/ml	2.51 ± 10.96	5.48 ± 17.86	0.72 ± 0.44	0.320
NSE, μg /L	20.10 ± 8.59	25.11 ± 10.16	17.10 ± 5.89	0.003

*AST, aspartate transaminase; ALT, alanine transaminase; LDH, lactate dehydrogenase; Alb, albumin; Glb, globulin; CK, creatine kinase; GFR, glomerular filtration rate; BUN, blood urea nitrogen; Cr, creatinine; ESR, erythrocyte sedimentation rate; CRP, C-reactive protein; FER, ferritin; CEA, carcino-embryonic antigen; CYFRA, cytokeratin 19 fragment; SCCA, squamous cell carcinoma antigen; NSE, neuron-specific enolase*.

**Table 4:** Immunologic tests for MDA5^+^DM-ILD.

**Characteristics**	**Total (*N* = 63)**	**Not improved (*n* = 19)**	**Improved (*n* = 44)**	***P*-value**
**Anti-MDA5**				0.024
Weakly positive, *n* (%)	7 (11.1)	0 (0.0)	7 (15.9)	
Positive, *n* (%)	25 (39.7)	11 (57.9)	14 (31.8)	
Strongly positive, *n* (%)	31 (49.2)	8 (42.1)	23 (52.3)	
CD3^+^ T, %	72.46 ± 12.13	70.15 ± 13.84	73.46 ± 11.37	0.367
CD4^+^ T, %	46.70 ± 13.19	48.04 ± 15.87	46.12 ± 12.05	0.630
CD8^+^ T, %	23.14 ± 11.19	19.41 ± 10.15	24.75 ± 11.36	0.112
B lymphocyte, %	17.54 ± 9.71	17.26 ± 8.54	17.67 ± 10.33	0.891
NK lymphocyte, %	7.95 ± 7.72	9.88 ± 11.59	7.04 ± 4.98	0.230
IL-2, pg/ml	2.62 ± 1.26	2.74 ± 1.01	2.57 ± 1.36	0.657
IL-4, pg/ml	2.59 ± 1.11	2.77 ± 0.98	2.52 ± 1.16	0.458
IL-6, pg/ml	39.76 ± 92.17	30.61 ± 54.40	43.71 ± 104.81	0.639
IL-10, pg/ml	5.77 ± 3.01	6.24 ± 3.51	5.57 ± 2.82	0.475
TNFα, pg/ml	10.56 ± 26.26	3.08 ± 1.77	13.67 ± 30.82	0.047
IFNγ, pg/ml	2.61 ± 1.39	2.54 ± 1.48	2.64 ± 1.38	0.809

*IL, interleukin; TNF, tumor necrosis factor; IFN, interferon*.

### Association Between Clinical Characteristics and Prognosis

The clinical characteristics of the patients with MDA5^+^ DM-ILD are summarized in Tables 1–4, Supplementary Table 1 according to basic information, pulmonary examinations, general laboratory tests, and immunologic tests based on their prognosis in the 400-day follow-up. CT scores of each lobe assessed by fibrotic-liking changes including GGO score, consolidation score, reticular score, and fibrosis score are shown in Supplementary Table 2.

**Figure 1:**
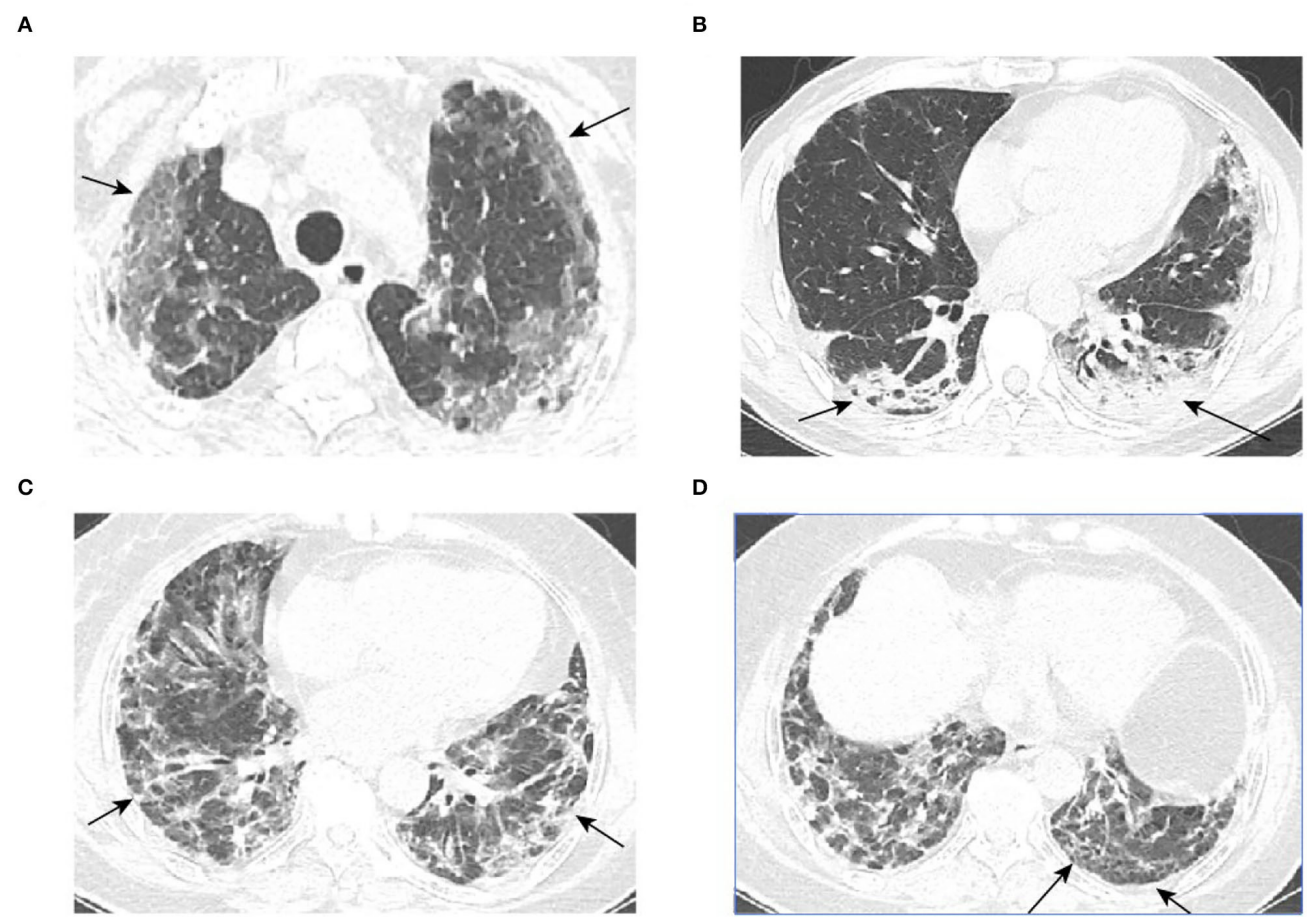
Representative CT images. **(A–D)** are typical GGO, consolidation, reticular, and honeycomb images, respectively (red arrows).

Previous research determined that prognosis was poor in elderly patients with MDA5^+^ DM-ILD (12). In our study, poor prognosis was found more frequently in acute-onset patients (2.87 ± 3.5 vs. 9.97 ± 16.85, *P* = 0.01). Abnormal symptoms such as fever (94.7% vs.36.4%, *P* < 0.001) and some complications such as pulmonary infection (100.0% vs. 36.4%, *P* < 0.001) and pleural effusion (73.7% vs. 20.5%, *P* < 0.001) were significantly associated with high mortality. The Gottron sign, skin ulceration, and heliotrope rash are characteristic cutaneous phenotypes in patients with MDA5^+^ DM and are significantly associated with increased risk of subacute ILD or RP-ILD (6, 26, 27). However, our results did not find that the signs above had an apparent link with prognosis of patients with MDA5^+^ DM-ILD. All the 63 patients here were not diagnosed with any internal malignancy. Despite many scholars suggesting MDA5^+^ DM is likely to complicate malignancy, malignancy is uncommon in MDA5^+^ DM-ILD, with an incidence of <5% (15, 28). As expected, TCTs, GGO score, consolidation score, reticular score, and fibrosis score were higher in patients with poor prognosis. Representative CT images of GGO, consolidation, and reticular and fibrotic changes are shown in Figure 1. In contrast, the value of FVC and FEV1 was lower in poor prognosis. It had been noted the severely affected pulmonary function especially the baseline FVC% was validated to be the most significant prognostic factor to predict the 6-month all-cause mortality based on a multi-center MDA5^+^ DM-ILD data with a cutoff value of 50%, which means mortality being 15% while FVC% >50% and mortality being 70% while FVC% <50% (29, 30).

A new AI algorithm-based analysis suggested that “MDA5 score” may serve as an applicable prognostic predictor for MDA5^+^ DM-ILD (31). Regarding the laboratory examination indicators in our research, we found that poor prognosis patients had more positive and strongly positive anti-MDA5 results (*P* = 0.024) than the survivors. Research studies have mentioned that predictive cytokines and chemokines including IL-6, IL-8, IL-10, IL-15, IL-18, TNFα, IFN-α, IP-10, and CX3CL1 had high levels in MDA5^+^ DM-ILD (32–35), especially CX3CL1, which was identified as involved in the pathogenesis of MDA5^+^ DM-ILD with a strong correlation of *r* = 0.89 between anti-MDA5 titer and CX3CL1.

Early and intensive immunomodulatory therapy has some effects on clinical parameters such as cytokines, antibodies, and hyperferritinemia and may lead to better prognosis of concomitant ILD (29). Nakashima et al. (36) reported that combined immunosuppressive therapy markedly improved the prognosis from 28.6 to 75%. An existing report revealed that the application of non-invasive positive pressure ventilation was an independent risk factor for survival (37). Based on this study, we were surprised to find that patients who received a larger dose of glucocorticoid (326.32 ± 284.65 vs. 86.77 ± 105.02, *P* = 0.002), antibiotic therapy (100% vs. 65.9%, *P* = 0.01), antiviral therapy (57.9% vs. 13.6%, *P* < 0.001), and NPPV (63.2% vs. 0%, *P* < 0.001) were more inclined to suffer a bad end. We had to owe poor prognosis after receiving intensive therapies to their complex and severe status liking secondary multiple infections. Although a previous clinical trial suggested that pirfenidone, in addition to conventional immunosuppressive treatment, did not result in improvement in terms of survival (38). We wanted to see if there is anti-fibrosis benefit. However, contrary to our expectations, the results showed that anti-fibrosis therapy did not improve the outcomes, maybe because the population incorporated was small and the follow-up was short.

Besides, we specially analyzed the correlation between the above clinical characteristics showing significant differences with prognosis and the survival time in the 400-day follow-up through Pearson correlation coefficient (Table 5). Majority of the results were consistent with those aforementioned.

**Table 5:** Correlation analysis of clinical characteristics and prognosis.

**Characteristics**	**Prognosis**		**Survival time**	
	***Pearson***	***P*-value**	***Pearson***	***P*-value**
Age	0.424	0.001	−0.365	0.006
CADM	0.317	0.011	−0.293	0.028
Duration	−0.226	0.075	0.294	0.028
Interval of DM and ILD	−0.210	0.099	0.258	0.054
Length of stay	0.408	0.001	−0.414	0.002
Fever	0.537	0.000	−0.509	0.000
Dyspnea	0.426	0.000	−0.379	0.004
Arthralgia	−0.302	0.016	0.272	0.043
Pulmonary infection	0.588	0.000	−0.538	0.000
Pleural effusion	0.507	0.000	−0.526	0.000
TCTs	0.491	0.000	−0.385	0.003
GGO score	0.337	0.007	−0.223	0.099
Consolidation score	0.565	0.000	−0.479	0.000
Reticular score	0.295	0.020	−0.297	0.026
Fibrosis score	0.291	0.022	−0.170	0.211
FVC	−0.305	0.075	0.276	0.126
FEV1	−0.280	0.103	0.262	0.148
Anti-MDA5	0.039	0.762	−0.051	0.709
Leukocyte	0.246	0.052	−0.279	0.038
Monocyte	0.426	0.001	−0.482	0.000
Neutrophil	0.239	0.059	−0.279	0.038
Proteinuria	0.313	0.017	−0.293	0.035
AST	0.307	0.014	−0.407	0.002
LDH	0.398	0.001	−0.378	0.004
Alb	−0.472	0.000	0.482	0.000
A/G	−0.307	0.014	0.273	0.042
GFR	−0.305	0.025	0.227	0.124
CRP	0.361	0.005	−0.353	0.010
FER	0.353	0.009	−0.254	0.072
TNFα	−0.186	0.192	0.181	0.224
CEA	0.217	0.142	−0.221	0.141
CYFRA	0.367	0.020	−0.320	0.047
NSE	0.217	0.142	−0.459	0.003
Glucocorticoid	0.532	0.000	−0.410	0.002
Antibiotic	0.367	0.003	−0.386	0.003
Antiviral	0.458	0.000	−0.366	0.006
NPPV	0.738	0.000	−0.649	0.000

### Prediction of the Prognosis of MDA5^+^DM-ILD

**Figure 2:**
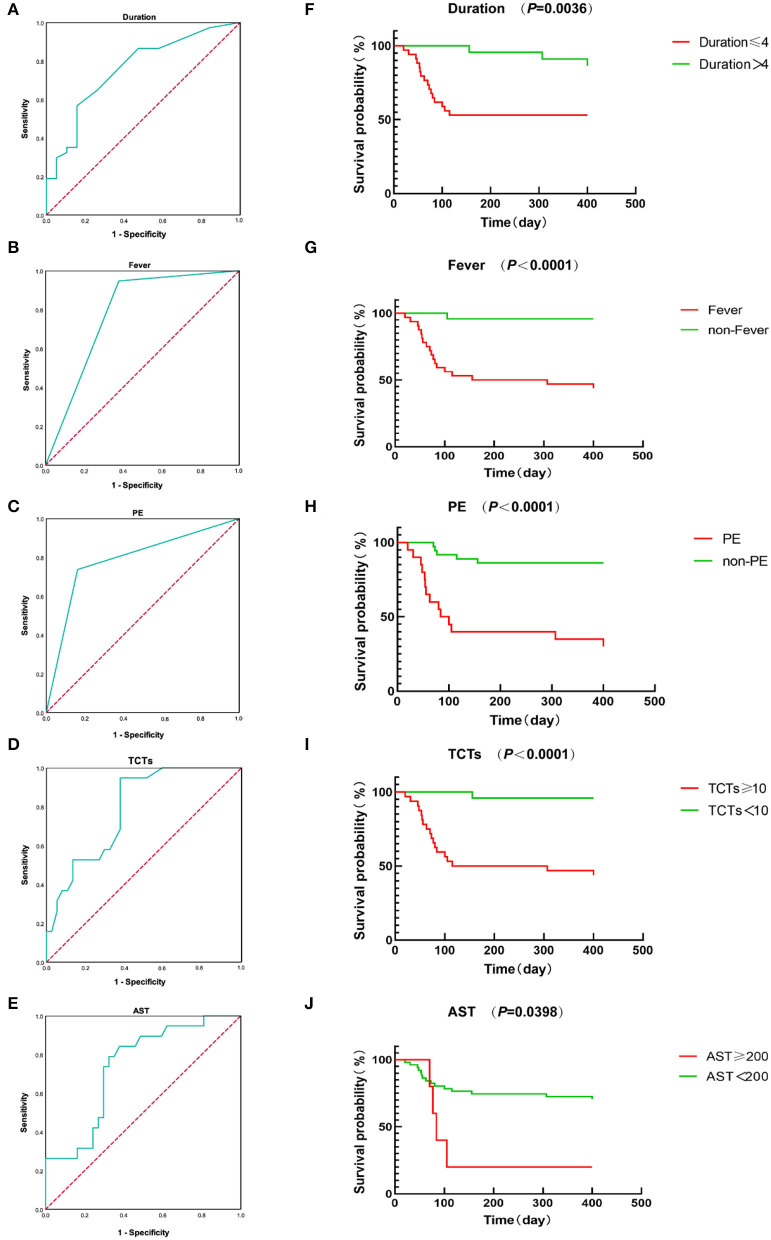
**(A–E)** ROC curves of duration, fever, PE, TCTs, and AST. **(F–J)** Kaplan-Meier survival curves of duration, fever, PE, TCTs, and AST.

The above studies have revealed some significant differences and associations between clinical characteristics and prognosis. Based on them, we next performed a univariate Cox regression analysis. Although there were many significant indicators included in our research, we selected only seven of them for the univariate Cox regression analysis following the rules of statistics (one indicator for 10 observations). As seen in Table 6, the univariate Cox regression analysis showed that fever, pulmonary infection, pleural effusion, TCTs, AST, and FER were significantly correlated with the prognosis of MDA5^+^DM-ILD. Then, inclusion of these factors and duration together in the multivariate Cox regression analysis revealed that duration, fever, PE, TCTs, and AST remained independent variables for predicting the prognosis of MDA5^+^DM-ILD. That is to say, acute onset (HR 0.827, *P* = 0.011), fever (HR 17.486, *P* = 0.012), pleural effusion (HR 0.174, *P* = 0.001), high TCTs (HR 1.048, *P* = 0.011), and high AST (HR 1.005, *P* = 0.004) were significant predictors of poor prognosis for MDA5^+^DM-ILD. Additionally, an ROC curve analysis was conducted to evaluate the predictive value of these factors (Table 7 and Figures 2A–E).

**Table 6:** Cox regression analysis of various predictive factors for the prognosis of MDA5^+^ DM-ILD.

**Characteristics**	**Univariate HR (95% CI)**	***P*-value**	**Multivariate HR (95% CI)**	***P*-value**
Duration		0.056	0.827 (0.704–0.972)	0.011
Fever	0.052 (0.007–0.388)	0.004	17.486 (1.861–164.255)	0.012
Pulmonary infection	61.142 (1.494–2,501.471)	0.030		
Pleural effusion	8.061 (2.884–22.532)	0.000	0.174 (0.059–0.511)	0.001
TCTs	1.049 (1.020–1.078)	0.001	1.048 (1.011–1.086)	0.011
AST	1.005 (1.002–1.008)	0.001	1.005 (1.002–1.009)	0.004
FER	1.000 (1.000–1.001)	0.032		

**Table 7:** ROC analysis of duration, fever, PE, TCTs, and AST.

**Characteristics**	**AUC**	**Youden index**	**95% CI**	**Sensitivity**	**Specificity**	***P*-value**
Duration	0.757	0.410	0.623–0.890	56.8%	84.2%	0.002
Fever	0.784	0.569	0.664–0.905	94.7%	62.2%	0.001
PE	0.787	0.575	0.652–0.923	73.7%	83.8%	0.000
TCTs	0.788	0.569	0.671–0.905	94.7%	62.2%	0.000
AST	0.738	0.465	0.604–0.871	78.9%	67.6%	0.004
Combination	0.954	0.788	0.902–1.000	84.2%	94.6%	0.000

*Combination: the combination of fever, PE, TCTs, and AST*.

### Development of Prognostic Nomogram Models of MDA5^+^DM-ILD

**Figure 3:**
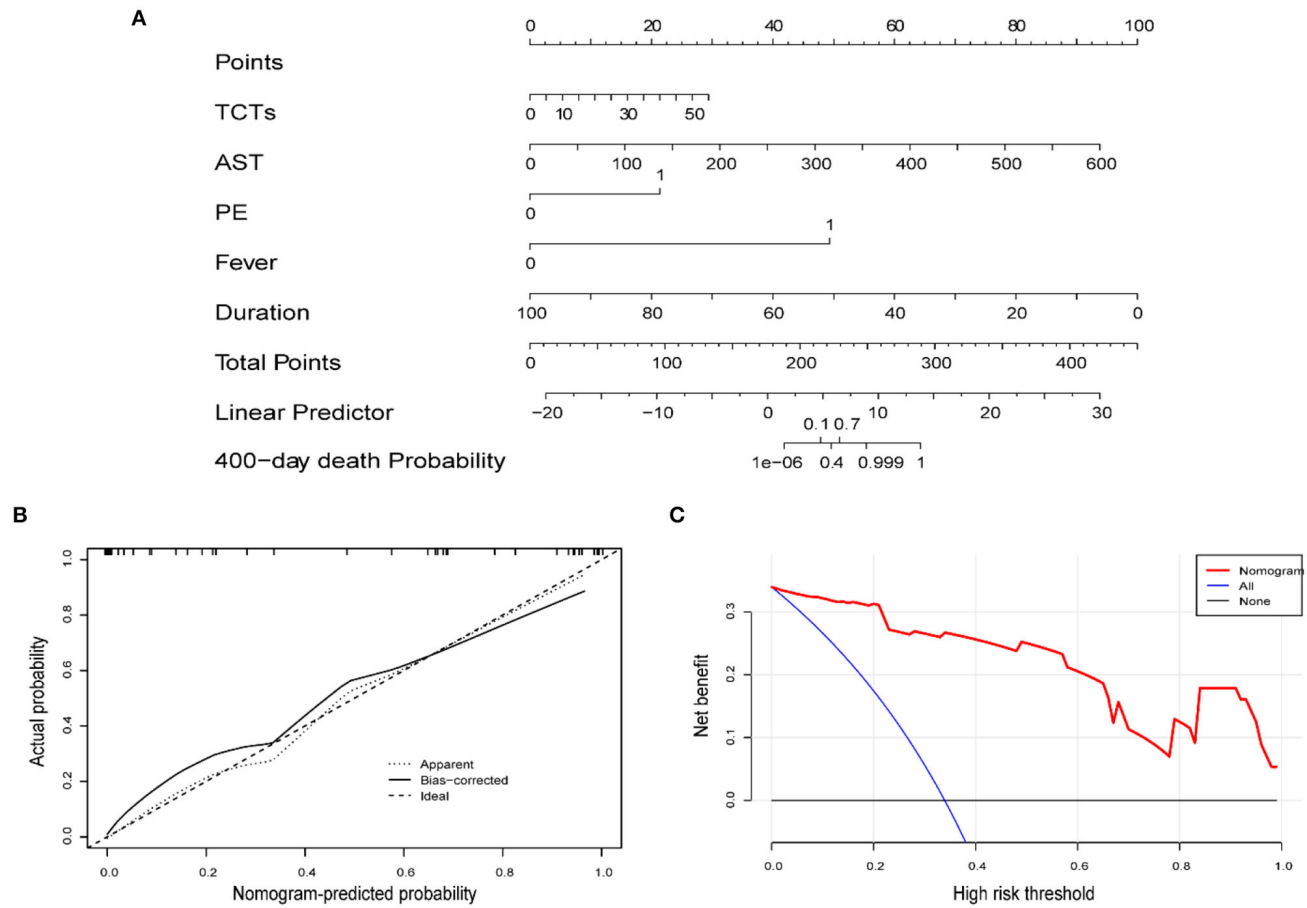
**(A)** Nomogram predicting the prognosis of MDA5^+^DM-ILD. **(B)** Calibration curve of the nomogram. **(C)** DCA of the nomogram.

A nomogram to predict the mortality of MDA5^+^DM-ILD was preliminarily constructed on the basis of multivariate Cox regression results (Figure 3A). Particularly, the nomogram was generated by assigning a weighed score on the point scale to each independent predictor. A higher score calculated from the sum of the assigned number of points for each prognostic parameter in the nomogram corresponds to a higher likelihood of death. The calibration curve showed that this predictive nomogram exhibited good calibration (Figure 3B). Moreover, a decision curve analysis (DCA) was conducted to assess the clinical utility of the predictive nomogram in Figure 3C.

**Figure 4:**
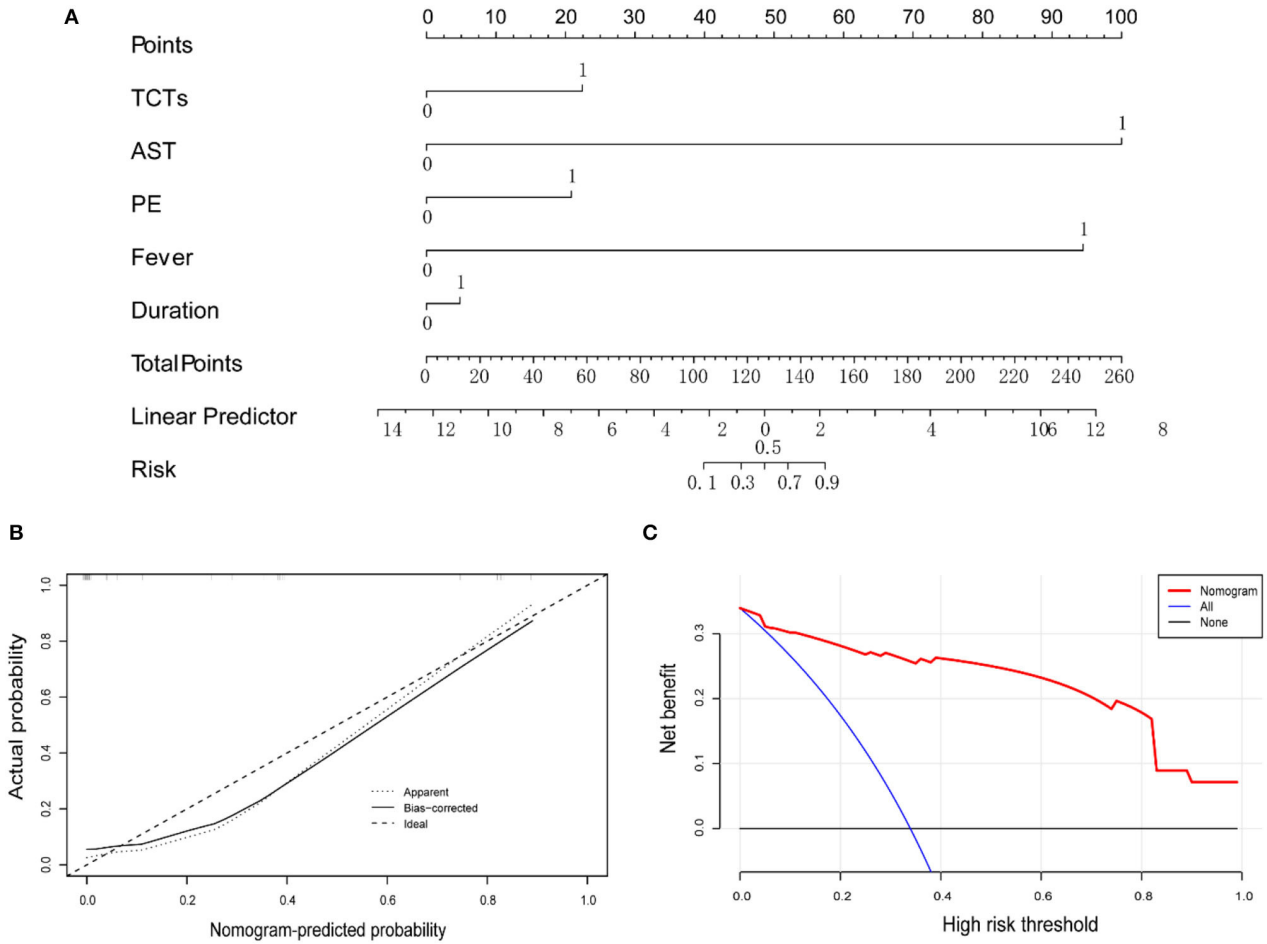
**(A)** Nomogram predicting the prognosis of MDA5^+^DM-ILD, while all predictors are binary variables. **(B)** Calibration curve of the nomogram. **(C)** DCA of the nomogram.

**Figure 5:**
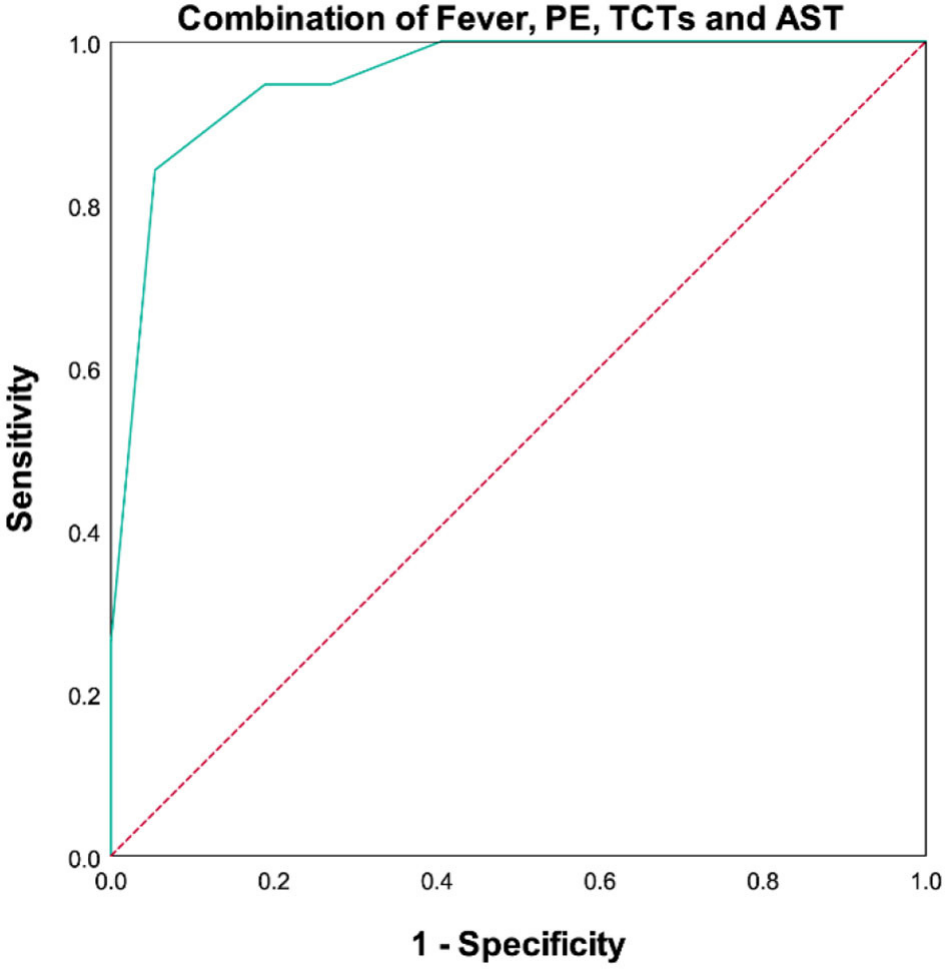
ROC curves of combination of fever, PE, TCTs, and AST.

To make this predictive model more convenient for physicians to use in clinical practice, we modified three predictors (duration, TCTs, and AST) into binary variables. Then three transformed binary variables together with fever and PE were used to conduct another nomogram model, in which all five predictors were evaluated with specific integer points: duration ≤ 4 m (5 points), fever (88 points), PE (21 points), TCTs ≥10 points (22 points), and AST ≥200 U/L (100 points) (Figures 4A–C). Then, we obtained Kaplan-Meier survival curves subdivided by duration ≤ 4 mouths, fever, PE, TCTs ≥10 points, and AST ≥200 U/L (Figures 2F–J). In the end, we created a new indicator by combining fever, PE, TCTs, and AST, which possessed good predictive ability, as reflected by an AUC of.954 (Table 7 and Figure 5).

## Discussion

The presence of MDA5^+^ DM-ILD can seriously impair the quality of life and shorten the survival of patients. The 6-month mortality of patients with MDA5^+^ DM-ILD ranges from 33 to 66% (10, 15, 16). A multicenter observational study (39) from 37 medical centers including 121 patients showed that MDA5^+^ RP-ILD had a noteworthy high mortality rate. Early and intensive immunomodulatory therapy has some effects on clinical parameters such as cytokines, antibodies, and hyperferritinemia, and may lead to better prognosis of concomitant ILD (29). Nakashima et al. (36) reported that combined immunosuppressive therapy markedly improved the prognosis from 28.6 to 75%. An existing report revealed that application of NPPV was an independent risk factor for survival (37). Previous studies on the predictive role of clinical characteristics for patients with MDA5^+^DM-ILD were relatively limited. Our study aimed to design a novel quantitative tool so clinicians can predict the probability of death. Thus, we integrated a total of 122clinical characteristics, 46 of which are shown in Supplementary Table 1. Although numerous clinical features were associated with prognosis, the clinical significance of a single index in the prediction of prognosis was quite limited because of one-sidedness. As a result, we selected the independent variables duration, fever, PE, TCTs, and AST, all being routine clinical practice, based on the multivariate Cox regression analysis to construct a predictive model.

Nakashima et al. (40) determined that the prognosis was poor in MDA5^+^DM-ILD patients who went through a long interval from appearance of skin lesions to diagnosis of ILD. Our data indicated the average course of disease and interval of DM and ILD in poor prognosis patients was 2.87 and 1.16 months, respectively, meaning acute onset of DM and ILD and serious, fractious conditions. Tanizawa et al. (14) also indicated that high fever was associated with poor prognosis of DM-ILD. Pleural effusion (73.7% vs. 20.5%, *P* < 0.001) was significantly associated with high mortality in this research. We systematically evaluated every patient's CT imaging and made points according to standard as mentioned above, finding the poor prognosis population getting visibly higher points not only on TCTs but also on GGO score, consolidation score, reticular score, and fibrosis score. It was reported that consolidation, GGO, and reticular opacities were distinctive findings in high-resolution computed tomography (HRCT) (14, 41) and that an initial right middle lobe GGO score of ≥2 (GGO ≥5% of the lobe) was a poor prognostic factor (42) for patients with MDA5^+^DM-ILD. Besides, a semi-quantitative HRCT scoring method including GGO, consolidation, and fibrosis was applied for the assessment of MDA5^+^ DM-ILD and confirmed an independent risk factor for 1-year mortality (43). However, the fibrosis components were heavily weighted in this scoring method. Recent research studies including an AI algorithm-based analysis named “AI score” revealed that lower zone GGO and consolidation demonstrated to be correlated with RP-ILD and were applicable prognostic predictors for MDA5^+^ DM-ILD (31, 44). Besides, the scores of microhemorrhage, capillary disorganization, spontaneous pneumomediastinum, and neoangiogenesis were significantly correlated with known poor prognosis factors of DM-ILD and total fibrosis scores of chest HRCT (37, 45, 46). Some research studies (9, 29, 37, 40, 47) have reported that anti-MDA5-positive and non-survivors presented higher serum AST level.

We can believe that each enrolled index in our model has a definite guiding function and an undoubted effect on clinic work. However, this model was generated in a specific patient population and specific clinical characteristics. Inevitably, this model may not be the standard model that represents all patients with MDA5^+^ DM-ILD and covers all possible clinical indicators. What we can do is to build a model that is as comprehensive and reliable as possible under existing conditions. Therefore, we suggest that one flaw of our model is that hyperferritinemia was not included. In fact, hyperferritinemia has been indicated as a key risk factor for patients with MDA5^+^ DM and RP-ILD (1, 10, 48–51). It is just that our model dropped it in the fitting process for some reason. Nevertheless, non-hyperferritinemia in the model does not mean that hyperferritinemia is not important, and it absolutely can be an independent prognostic factor.

Different predictive models have been reported in the past 10 years. “FLAIR score,” including ferritin, LDH, semi-quantitative anti-MDA5 grade, HRCT imaging score, and RPILD/non-RPILD based on a large-scale Chinese single-center cohort (*n* = 207), was proposed to predict mortality in CADM-ILD (1). Other reports also stated that ferritin, LDH, and KL-6 were independent high-risk factors for poor outcomes (1, 52, 53). A multivariate logistic regression analysis (27) previously indicated that positive anti-MDA5, elevated CRP, and decreased counts of lymphocyte can provide a precise prediction for RP-ILD in patients with CADM. The evidence-based risk prediction model using CRP and KL-6 combined with anti-MDA5 might also be useful for predicting prognosis in patients with DM-ILD; it is called the MCK (MDA5, CRP, and KL-6) model, identifying patients at low (<15%), moderate (15–49%), or high risk (≥50%) of mortality based on the number of risk factors. Respiratory physiological parameters such as lower arterial partial pressure of oxygen (PaO2) and higher alveolar-arterial oxygen difference (AaDO2) have been associated with the development of RPILD and poor prognosis in several small-sample MDA5^+^ DM/CADM studies (10, 42). Unfortunately, the heterogeneity of these cohorts was obvious, and the pulmonary function and structure evaluation were suboptimal.

This is the first time that duration, fever, PE, TCTs, and AST are recommended together as a predictor for the prognosis of MDA5^+^ DM-ILD. This nomogram has high predictive accuracy and can be applied in most hospitals because of convenience. With the aim of establishing a novel scoring system, we converted the nomogram into a scoring system. If the total score is over 116 points, a high probability (≥30%) of mortality exists. Meanwhile, when we combined fever, PE, TCTs, and AST together, a nice predictive function can be seen: AUC being.954, sensitivity being 84.2%, and specificity being 94.6% on the ROC curve. Hence, this method is not only feasible and simple but could also accurately recognize poor prognosis with high calibration.

This study is not exempt from limitations. First, this study was based on retrospective data, and the validity of the retrospective data was limited. Moreover, the size of the sample included in this study was small. Next, the nomogram model was not validated in the external validation set from other medical centers. Finally, our follow-up time was relative short, lacking assessment of long-term survival conditions. Therefore, multicenter validation of the scoring system with a large study population is urgently needed to obtain high-level evidence for its clinical application in the future.

In conclusion, the predictive model for the prognosis of MDA5^+^ DM-ILD assists in identifying cases accurately, intensifying treatment early, and saving as many patient lives as possible in clinical practice. This study is based on a unicentric and small sample of participants suggesting a favorable predictive performance and should be further validated in multicenter prospective studies in the near future.

## Data Availability Statement

The datasets presented in this article are not readily available because the data are being expanded for use in another study. Requests to access the datasets should be directed to 476839887@qq.com.

## Ethics Statement

Written informed consent was obtained from the individual(s) for the publication of any potentially identifiable images or data included in this article.

## Author Contributions

QN and L-qZ conceived the study, collected the data, and performed the analysis. QN wrote the manuscript. W-lM, LX, X-rW, X-lH, and FY made suggestions on the revision of manuscript. All authors contributed to the article and approved the submitted version.

## Conflict of Interest

The authors declare that the research was conducted in the absence of any commercial or financial relationships that could be construed as a potential conflict of interest.

## Publisher's Note

All claims expressed in this article are solely those of the authors and do not necessarily represent those of their affiliated organizations, or those of the publisher, the editors and the reviewers. Any product that may be evaluated in this article, or claim that may be made by its manufacturer, is not guaranteed or endorsed by the publisher.

## References

[1] Lian X Zou J Guo Q Chen S Lu L Wang R. Mortality risk prediction in amyopathic dermatomyositis associated with interstitial lung disease: the FLAIR model. Chest. (2020) 158:1535–45. 10.1016/j.chest.2020.04.057 32428508

[2] Kiely PD Chua F. Interstitial lung disease in inflammatory myopathies: clinical phenotypes and prognosis. Curr Rheumatol Rep. (2013) 15:359. 10.1007/s11926-013-0359-6 23888366

[3] Takeuchi O Akira S. MDA5/RIG-I and virus recognition. Curr Opin Immunol. (2008) 20:17–22. 10.1016/j.coi.2008.01.002 18272355

[4] Kato H Takeuchi O Mikamo-Satoh E Hirai R Kawai T Matsushita K. Length-dependent recognition of double-stranded ribonucleic acids by retinoic acid-inducible gene-I and melanoma differentiation-associated gene 5. J Exp Med. (2008) 205:1601–10. 10.1084/jem.20080091 18591409 PMC2442638

[5] Sato S Hirakata M Kuwana M Suwa A Inada S Mimori T. Autoantibodies to a 140-kd polypeptide, CADM-140, in Japanese patients with clinically amyopathic dermatomyositis. Arthritis Rheum. (2005) 52:1571–6. 10.1002/art.21023 15880816

[6] Fiorentino D Chung L Zwerner J Rosen A Casciola-Rosen L. The mucocutaneous and systemic phenotype of dermatomyositis patients with antibodies to MDA5 (CADM-140): a retrospective study. J Am Acad Dermatol. (2011) 65:25–34. 10.1016/j.jaad.2010.09.016 21531040 PMC3167687

[7] Sato S Kuwana M Fujita T Suzuki Y. Anti-CADM-140/MDA5 autoantibody titer correlates with disease activity and predicts disease outcome in patients with dermatomyositis and rapidly progressive interstitial lung disease. Mod Rheumatol. (2012) 23:496–502. 10.1007/s10165-012-0663-4 22644102

[8] Hall JC Casciola-Rosen L Samedy LA Werner J Owoyemi K Danoff SK. Anti-melanoma differentiation-associated protein 5-associated dermatomyositis: expanding the clinical spectrum. Arthritis Care Res. (2013) 65:1307–15. 10.1002/acr.21992 23436757 PMC3689861

[9] Chen F Wang D Shu X Nakashima R Wang G. Anti-MDA5 antibody is associated with A/SIP and decreased T cells in peripheral blood and predicts poor prognosis of ILD in Chinese patients with dermatomyositis. Rheumatol Int. (2012) 32:3909–15. 10.1007/s00296-011-2323-y 22198664

[10] Gono T Sato S Kawaguchi Y Kuwana M Hanaoka M Katsumata Y. Anti-MDA5 antibody, ferritin and IL-18 are useful for the evaluation of response to treatment in interstitial lung disease with anti-MDA5 antibody-positive dermatomyositis. Rheumatology. (2012) 51:1563–70. 10.1093/rheumatology/kes102 22589330

[11] Matsushita T Mizumaki K Kano M Yagi N Tennichi M Takeuchi A. Antimelanoma differentiation-associated protein 5 antibody level is a novel tool for monitoring disease activity in rapidly progressive interstitial lung disease with dermatomyositis. Br J Dermatol. (2017) 176:395–402. 10.1111/bjd.14882 27452897

[12] Li S Ge Y Yang M. Correlation analysis of myositis specific antibody spectrum and clinical features in 427 patients with dermatomyositis. Chin J Rheumatol. (2017) 21:585–94. 10.3760/cma.j.issn.1007-7480.2017.09.003

[13] Motegi SI Sekiguchi A Toki S Kishi C Endo Y Yasuda M. Clinical features and poor prognostic factors of anti-melanoma differentiation-associated gene 5 antibody-positive dermatomyositis with rapid progressive interstitial lung disease. Eur J Dermatol. (2019) 29:511–7. 10.1684/ejd.2019.3634 31617496

[14] Tanizawa K Handa T Nakashima R Kubo T Hosono Y Watanabe K. HRCT features of interstitial lung disease in dermatomyositis with anti-CADM-140 antibody. Respir Med. (2011) 105:1380–7. 10.1016/j.rmed.2011.05.006 21632230

[15] Koga T Fujikawa K Horai Y Okada A Kawashiri SY Iwamoto N. The diagnostic utility of anti-melanoma differentiation-associated gene 5 antibody testing for predicting the prognosis of Japanese patients with DM. Rheumatology. (2012) 51:1278–84. 10.1093/rheumatology/ker518 22378718

[16] Tsuji H Nakashima R Hosono Y Imura Y Yagita M Yoshifuji H. Multicenter prospective study of the efficacy and safety of combined immunosuppressive therapy with high-dose glucocorticoid, tacrolimus, and cyclophosphamide in interstitial lung diseases accompanied by anti-melanoma differentiation-associated gene 5-positive dermatomyositis. Arthritis Rheumatol. (2020) 72:488–98. 10.1002/art.41105 31524333

[17] Gono T Kawaguchi Y Ozeki E Ota Y Satoh T Kuwana M. Serum ferritin correlates with activity of anti-MDA5 antibody-associated acute interstitial lung disease as a complication of dermatomyositis. Mod Rheumatol. (2011) 21:223–7. 10.3109/s10165-010-0371-x 21052763

[18] Bohan A Peter JB. Polymyositis and dermatomyositis. N Engl J Med. (1975) 292:344–7. 10.1056/NEJM197502132920706 1090839

[19] Bohan A Peter JB. Polymyositis and dermatomyositis. N Engl J Med. (1975) 292:403–7. 10.1056/NEJM197502202920807 1090839

[20] Sontheimer RD. Would a new name hasten the acceptance of amyopathic dermatomyositis (dermatomyositis sine myositis) as a distinctive subset within the idiopathic inflammatory dermatomyopathies spectrum of clinical illness? J Am Acad Dermatol. (2002) 46:626–36. 10.1067/mjd.2002.120621 11907524

[21] Hansell DM Bankier AA MacMahon H McLoud TC Müller NL Remy J. Fleischner society: glossary of terms for thoracic imaging. Radiology. (2008) 246:697–722. 10.1148/radiol.2462070712 18195376

[22] Antonio GE Wong KT Hui DS Wu A Lee N Yuen EH. Thin-section CT in patients with severe acute respiratory syndrome following hospital discharge: preliminary experience. Radiology. (2003) 228:810–5. 10.1148/radiol.2283030726 12805557

[23] Xu W Wu W Zheng Y Chen Z Tao X Zhang D. A computed tomography radiomics-based prediction model on interstitial lung disease in anti-MDA5-positive dermatomyositis. Front Med. (2021) 8:768052. 10.3389/fmed.2021.768052 34912828 PMC8667862

[24] Ruaro B Baratella E Confalonieri P Wade B Marrocchio C Geri P. High-resolution computed tomography: lights and shadows in improving care for SSc-ILD patients. Diagnostics. (2021) 11:1960. 10.3390/diagnostics11111960 34829307 PMC8617987

[25] Chang YC Yu CJ Chang SC Galvin JR Liu HM Hsiao CH. Pulmonary sequelae in convalescent patients after severe acute respiratory syndrome: evaluation with thin-section CT. Radiology. (2005) 236:1067–75. 10.1148/radiol.2363040958 16055695

[26] Cao H Xia Q Pan M Zhao X Li X Shi R. Gottron papules and Gottron sign with ulceration: a distinctive cutaneous feature in a subset of patients with classic dermatomyositis and clinically amyopathic dermatomyositis. J Rheumatol. (2016) 43:1735–42. 10.3899/jrheum.160024 27307530

[27] Xu Y Yang CS Li YJ Liu XD Wang JN Zhao Q. Predictive factors of rapidly progressive-interstitial lung disease in patients with clinically amyopathic dermatomyositis. Clin Rheumatol. (2016) 35:113–6. 10.1007/s10067-015-3139-z 26660480

[28] So H Ip RW Wong VT Yip RM. Analysis of anti-melanoma differentiation-associated gene 5 antibody in Hong Kong Chinese patients with idiopathic inflammatory myopathies: diagnostic utility and clinical correlations. Int J Rheum Dis. (2018) 21:1076–81. 10.1111/1756-185X.13268 29380533

[29] Gono T Kawaguchi Y Satoh T Kuwana M Katsumata Y Takagi K. Clinical manifestation and prognostic factor in anti-melanoma differentiation-associated gene 5 antibody-associated interstitial lung disease as a complication of dermatomyositis. Rheumatology. (2010) 49:1713–9. 10.1093/rheumatology/keq149 20498012

[30] Wu W Xu W Sun W Zhang D Zhao J Luo Q. Forced vital capacity predicts the survival of interstitial lung disease in anti-MDA5 positive dermatomyositis: a multi-center cohort study. Rheumatology. (2021) 61:230–9. 10.1093/rheumatology/keab305 33764398

[31] Xu W Wu W Zhang D Chen Z Tao X Zhao J. A novel CT scoring method predicts the prognosis of interstitial lung disease associated with anti-MDA5 positive dermatomyositis. Sci Rep. (2021) 11:17070. 10.1038/s41598-021-96292-w 34426622 PMC8382835

[32] Gono T Kaneko H Kawaguchi Y Hanaoka M Kataoka S Kuwana M. Cytokine profiles in polymyositis and dermatomyositis complicated by rapidly progressive or chronic interstitial lung disease. Rheumatology. (2014) 53:2196–203. 10.1093/rheumatology/keu258 24970922

[33] Horai Y Koga T Fujikawa K Takatani A Nishino A Nakashima Y. Serum interferon-α is a useful biomarker in patients with anti-melanoma differentiation-associated gene 5 (MDA5) antibody-positive dermatomyositis. Mod Rheumatol. (2015) 25:85–9. 10.3109/14397595.2014.900843 24716595

[34] Takada T Ohashi K Hayashi M Asakawa K Sakagami T Kikuchi T. Role of IL-15 in interstitial lung diseases in amyopathic dermatomyositis with anti-MDA-5 antibody. Respir Med. (2018) 141:7–13. 10.1016/j.rmed.2018.06.012 30053975

[35] Chen M Quan C Diao L Xue F Xue K Wang B. Measurement of cytokines and chemokines and association with clinical severity of dermatomyositis and clinically amyopathic dermatomyositis. Br J Dermatol. (2018) 179:1334–41. 10.1111/bjd.17079 30101523

[36] Nakashima R Mimori T. Anti-MDA5 (melanoma differentiation-associated gene 5) antibody and dermatomyositis with rapidly progressive interstitial pneumonia. Nihon Rinsho Meneki Gakkai Kaishi. (2013) 36:711–6. 10.2177/jsci.36.71 23629426

[37] Zhou M Ye Y Yan N Lian X Bao C Guo Q. Non-invasive positive pressure ventilator deteriorates the outcome of pneumomediastinum in anti-MDA5 antibody-positive clinically amyopathic dermatomyositis. Clin Rheumatol. (2020) 39:1919–27. 10.1007/s10067-019-04918-2 31942657

[38] Li T Guo L Chen Z Gu L Sun F Tan X. Pirfenidone in patients with rapidly progressive interstitial lung disease associated with clinically amyopathic dermatomyositis. Sci Rep. (2016) 6:33226. 10.1038/srep33226 27615411 PMC5018967

[39] Allenbach Y Uzunhan Y Toquet S Leroux G Gallay L Marquet A. Different phenotypes in dermatomyositis associated with anti-MDA5 antibody: study of 121 cases. Neurology. (2020) 95:e70–8. 10.1212/WNL.0000000000009727 32487712 PMC7371381

[40] Nakashima R Imura Y Kobayashi S Yukawa N Yoshifuji H Nojima T. The RIG-I-like receptor IFIH1/MDA5 is a dermatomyositis-specific autoantigen identified by the anti-CADM-140 antibody. Rheumatology. (2009) 49:433–40. 10.1093/rheumatology/kep375 20015976

[41] Hozumi H Fujisawa T Nakashima R Johkoh T Sumikawa H Murakami A. Comprehensive assessment of myositis-specific autoantibodies in polymyositis/dermatomyositis-associated interstitial lung disease. Respir Med. (2016) 121:91–9. 10.1016/j.rmed.2016.10.019 27888997

[42] Fujiki Y Kotani T Isoda K Ishida T Shoda T Yoshida S. Evaluation of clinical prognostic factors for interstitial pneumonia in anti-MDA5 antibody-positive dermatomyositis patients. Mod Rheumatol. (2018) 28:133–40. 10.1080/14397595.2017.1318468 28490218

[43] Zou J Guo Q Chi J Wu H Bao C. HRCT score and serum ferritin level are factors associated to the 1-year mortality of acute interstitial lung disease in clinically amyopathic dermatomyositis patients. Clin Rheumatol. (2015) 34:707–14. 10.1007/s10067-015-2866-5 25609178

[44] Zuo Y Ye L Liu M Li S Liu W Chen F. Clinical significance of radiological patterns of HRCT and their association with macrophage activation in dermatomyositis. Rheumatology. (2020) 59:2829–37. 10.1093/rheumatology/keaa034 32065646

[45] Wakura R Matsuda S Kotani T Shoda T Takeuchi T. The comparison of nailfold videocapillaroscopy findings between anti-melanoma differentiation-associated gene 5 antibody and anti-aminoacyl tRNA synthetase antibody in patients with dermatomyositis complicated by interstitial lung disease. Sci Rep. (2020) 10:15692. 10.1038/s41598-020-72752-7 32973255 PMC7518258

[46] Yamaguchi K Yamaguchi A Itai M Kashiwagi C Takehara K Aoki S. Clinical features of patients with anti-melanoma differentiation-associated gene-5 antibody-positive dermatomyositis complicated by spontaneous pneumomediastinum. Clin Rheumatol. (2019) 38:3443–50. 10.1007/s10067-019-04729-5 31420814

[47] Hoshino K Muro Y Sugiura K Tomita Y Nakashima R Mimori T. Anti-MDA5 and anti-TIF1-γ antibodies have clinical significance for patients with dermatomyositis. Rheumatology. (2010) 49:1726–33. 10.1093/rheumatology/keq153 20501546

[48] Zuo Y Ye L Chen F Shen Y Lu X Wang G. Different multivariable risk factors for rapid progressive interstitial lung disease in anti-MDA5 positive dermatomyositis and anti-synthetase syndrome. Front Immunol. (2022) 13:845988. 10.3389/fimmu.2022.845988 35320936 PMC8936070

[49] Shirakashi M Nakashima R Tsuji H Tanizawa K Handa T Hosono Y. Efficacy of plasma exchange in anti-MDA5-positive dermatomyositis with interstitial lung disease under combined immunosuppressive treatment. Rheumatology. (2020) 59:3284–92. 10.1093/rheumatology/keaa123 32276271

[50] Wang LM Yang QH Zhang L Liu SY Zhang PP Zhang X. Intravenous immunoglobulin for interstitial lung diseases of anti-melanoma differentiation-associated gene 5-positive dermatomyositis. Rheumatology. (2021). 10.1093/rheumatology/keab928 [Epub ahead of print]. 34940809

[51] Koguchi-Yoshioka H Okiyama N Iwamoto K Matsumura Y Ogawa T Inoue S. Intravenous immunoglobulin contributes to the control of antimelanoma differentiation-associated protein 5 antibody-associated dermatomyositis with palmar violaceous macules/papules. Br J Dermatol. (2017) 177:1442–6. 10.1111/bjd.15499 28346662

[52] Kurasawa K Arai S Namiki Y Tanaka A Takamura Y Owada T. Tofacitinib for refractory interstitial lung diseases in anti-melanoma differentiation-associated 5 gene antibody-positive dermatomyositis. Rheumatology. (2018) 57:2114–9. 10.1093/rheumatology/key188 30060040

[53] Bandoh S Fujita J Ohtsuki Y Ueda Y Hojo S Tokuda M. Sequential changes of KL-6 in sera of patients with interstitial pneumonia associated with polymyositis/dermatomyositis. Ann Rheum Dis. (2000) 59:257–62. 10.1136/ard.59.4.257 10733471 PMC1753113

