# Supplementary material for: A New Predictive Model for the Prognosis of MDA5+ DM-ILD
Source: Front Med (Lausanne). 2022 Jun 15;9:908365. doi: 10.3389/fmed.2022.908365 (PMC9240232; doi:10.3389/fmed.2022.908365)
Supplement: Supplementary file 1 [file Data_Sheet_1.PDF]

Supplemental Table 1 Clinical characteristics of MDA5$^+$ DM-ILD

Characteristics	Total (N=63)	Not improved (n=19)	Improved (n=44)	*P*-value
Acropachia, n (%)	1 (1.6)	0 (0.0)	1 (2.3)	1.000
Pneumothorax, n (%)	0 (0.0)	0 (0.0)	0 (0.0)	
Erythrocyte, T/L	3.90±0.45	3.89±0.50	3.90±0.44	0.941
Hemoglobin, g/L	114.75±14.55	112.32±13.06	115.80±15.17	0.388
Platelet, G/L G/L	202.08±74.67	208.79±88.52	199.18±68.77	0.643
Eosinophil, G/L	0.07±0.13	0.08±0.19	0.06±0.10	0.742
Eosinophil, %	1.21±1.85	1.00±2.25	1.31±1.67	0.548
Basophil, G/L	0.02±0.02	0.02±0.03	0.02±0.01	0.485
Basophil, %	0.29±0.23	0.24±0.19	0.32±0.25	0.265
FIB, g/L	3.94±1.24	4.07±1.57	3.88±1.07	0.586
APTT, s	39.09±6.36	39.86±7.38	38.75±5.92	0.544
INR	1.01±0.19	0.99±0.12	1.01±0.22	0.626
PT, s	12.98±1.87	12.79±1.24	13.07±2.10	0.603
D dimer, mg/L FEU	1.64±2.73	2.62±4.45	1.12±0.71	0.175
FDP, µg/ml	4.83±3.58	6.15±4.61	3.97±2.47	0.107
ATIII, %	89.11±17.67	85.53±21.76	91.43±14.47	0.365
TT, s	18.32±1.60	18.81±1.81	18.10±1.47	0.119
TBIL, U/L	10.66±5.60	10.39±5.08	10.77±5.86	0.810
ALP, U/L	95.52±112.36	121.95±160.51	84.11±83.53	0.223
GGT, U/L	103.84±152.22	147.74±188.64	84.89±131.50	0.134
Total CO_2, mmol/L	25.09±2.59	24.42±3.12	25.38±2.30	0.179
Coxsackie virus B3				0.493
Negative, n (%)	44 (91.7)	13 (100.0)	31 (88.6)	
Positive, n (%)	4 (8.3)	0 (0.0)	4 (11.4)	
Coxsackie virus B5				1.000
Negative, n (%)	39 (81.3)	11 (84.6)	28 (80.0)	
Positive, n (%)	9 (18.7)	2 (15.4)	7 (20.0)	
Enterovirus				0.675
Negative, n (%)	30 (62.5)	7 (53.8)	23 (65.7)	
Positive, n (%)	18 (37.5)	6 (46.2)	12 (34.3)	
Cytomegalovirus				0.976
Negative, n (%)	46 (90.2)	13 (86.7)	33 (91.7)	
Positive, n (%)	5 (9.8)	2 (13.3)	3 (8.3)	
EB virus				0.289
Negative, n (%)	24 (57.1)	9 (69.2)	15 (51.7)	
Positive, n (%)	18 (42.9)	4 (30.8)	14 (48.3)	
CD4/CD8	2.88±2.47	3.70±3.48	2.52±1.83	0.214
ANA				0.888
Negative, n (%)	18 (34.0)	6 (35.3)	12 (33.3)	
Positive, n (%)	35 (66.0)	11 (64.7)	24 (66.7)	
Ro-52				0.889

Negative, n (%)	29 (46.0)	9 (47.4)	20 (45.5)	
Positive, n (%)	34 (54.0)	10 (52.6)	24 (54.5)	
Jo-1				0.547
Negative, n (%)	60 (95.2)	19 (100.0)	41 (93.2)	
Positive, n (%)	3 (4.8)	0 (0.0)	3 (6.8)	
RF				0.13
Negative, n (%)	47 (92.2)	12 (80.0)	35 (97.2)	
Positive, n (%)	4 (7.8)	3 (20.0)	1 (2.8)	
IgE, IU/ml	198.48±285.73	305.40±295.96	154.61±273.23	0.075
IgG, g/L	13.70±3.95	14.26±3.15	13.47±4.25	0.506
IgM, g/L	1.49±0.72	1.39±0.65	1.54±0.75	0.500
IgA, g/L	2.35±0.89	2.61±1.00	2.24±0.83	0.162
C3, g/L	0.76±0.17	0.71±0.21	0.78±0.15	0.171
C4, g/L	0.23±0.08	0.26±0.11	0.22±0.06	0.153
AFP, μg /L	3.30±2.62	3.27±2.12	3.32±2.90	0.954
CA125, U/ml	17.17±16.47	21.52±22.00	14.56±11.81	0.200
CA199, U/ml	35.57±133.21	71.55±216.01	13.98±17.90	0.320
CA153, U/ml	24.56±13.10	28.05±18.98	22.47±7.52	0.196
CA724, U/ml	4.07±6.19	4.08±7.78	4.06±5.20	0.992
HE4, pmol/L	121.17±48.23	136.21±46.02	111.91±48.96	0.273
HCG-β, IU/L	1.47±0.82	1.26±1.12	1.59±0.02	0.252
frPSA, μg /L	0.23±0.17	0.26±0.21	0.20±0.14	0.482
PSA, μg /L	0.93±0.79	1.12±0.97	0.81±0.67	0.422

FIB, fibrinogen; APTT, activated partial thromboplastin time; INR, international normalized ratio; PT, prothrombin time; FDP, fibrin degradation product; ATIII, antithrombin III; TT, thrombin time; TBIL, total bilirubin; ALP, alkaline phosphatase; GGT, gamma-glutamyl transpeptidase; ANA, antinuclear antibody; RF, rheumatoid factor; Ig, immune globulin; C, complement; AFP, alpha fetoprotein; CA, carbohydrate antigen; HE4, human epididymisprotein 4; HCG-β, human chorionic gonadotrophin-β; frPSA, free prostate-specific antigen; PSA, prostate-specific antigen.

Supplemental Table 2 CT scores of fibrotic-like imaging

Characteristics	Lobe	Total	Not improved	Improved	*P*
GGO score	Right upper	0.76±0.99	1.37±1.21	0.49±0.74	0.001
	Right middle	0.63±0.98	1.26±1.33	0.35±0.61	0.009
	Right lower	1.39±1.31	1.63±1.21	1.28±1.35	0.333
	Left upper	0.85±0.88	1.42±1.17	0.60±0.58	0.009
	Left lower	1.42±1.34	1.63±1.26	1.33±1.38	0.411
Consolidation score	Right upper	0.31±0.62	0.74±0.81	0.12±0.39	0.004
	Right middle	0.42±0.86	1.00±1.29	0.16±0.37	0.012
	Right lower	0.85±1.19	1.63±1.34	0.51±0.94	0.000
	Left upper	0.58±0.88	1.42±0.90	0.21±0.56	0.000
	Left lower	0.89±1.38	1.84±1.77	0.47±0.91	0.004
Reticular score	Right upper	0.24±0.59	0.37±0.76	0.19±0.50	0.267
	Right middle	0.34±0.72	0.74±0.99	0.16±0.49	0.025
	Right lower	1.02±1.32	1.47±1.54	0.81±1.18	0.070
	Left upper	0.21±0.55	0.37±0.76	0.14±0.41	0.230
	Left lower	0.95±1.25	1.32±1.46	0.79±1.13	0.127
Fibrosis score	Right upper	0.23±0.66	0.42±0.96	0.14±0.47	0.237
	Right middle	0.32±0.81	0.74±1.20	0.14±0.47	0.047
	Right lower	1.26±1.58	1.68±1.70	1.07±1.50	0.159
	Left upper	0.21±0.60	0.47±0.91	0.09±0.37	0.091
	Left lower	1.21±1.54	1.74±1.70	0.98±1.42	0.072
